# The eGFR-C study: accuracy of glomerular filtration rate (GFR) estimation using creatinine and cystatin C and albuminuria for monitoring disease progression in patients with stage 3 chronic kidney disease - prospective longitudinal study in a multiethnic population

**DOI:** 10.1186/1471-2369-15-13

**Published:** 2014-01-14

**Authors:** Edmund J Lamb, Elizabeth A Brettell, Paul Cockwell, Neil Dalton, Jon J Deeks, Kevin Harris, Tracy Higgins, Philip A Kalra, Kamlesh Khunti, Fiona Loud, Ryan S Ottridge, Claire C Sharpe, Alice J Sitch, Paul E Stevens, Andrew J Sutton, Maarten W Taal

**Affiliations:** 1 Clinical Biochemistry, East Kent Hospitals University NHS Foundation Trust, Canterbury, Kent CT1 3NG, UK; 2 Birmingham Clinical Trials Unit, School of Cancer Sciences, Robert Aitken Institute, University of Birmingham, Birmingham B15 2TT, UK; 3 University Hospitals Birmingham NHS Foundation Trust, Birmingham B15 2TT, UK; 4 Kings College London, London, UK; 5 Test Evaluation Research Group, School of Health and Population Sciences, Public Health Building, University of Birmingham, Birmingham, B15 2TT, UK; 6 University Hospitals of Leicester, Leicester, UK; 7 Centre for Health Services Studies, University of Kent, Canterbury CT2 7NF, UK; 8 Salford Royal NHS Foundation Trust, Salford M6 8HD, UK; 9 University of Leicester, Leicester, UK; 10 British Kidney Patient Association, Hampshire, UK; 11 Kent Kidney Care Centre, East Kent Hospitals University NHS Foundation Trust, Canterbury, Kent CT1 3NG, UK; 12 King’s College London & King’s College Hospital NHS Foundation Trust SE5 9RJ, London, UK; 13 Health Economics Unit, School of Health and Population Sciences, Occupational Health Building, University of Birmingham, Birmingham B15 2TT, UK; 14 Royal Derby Hospital, Uttoxeter Road, Derby DE22 3NE, UK

**Keywords:** Albuminuria, Biological variation, Creatinine, Cystatin C, Glomerular filtration rate, Iohexol, Kidney disease

## Abstract

**Background:**

Uncertainty exists regarding the optimal method to estimate glomerular filtration rate (GFR) for disease detection and monitoring. Widely used GFR estimates have not been validated in British ethnic minority populations.

**Methods/design:**

Iohexol measured GFR will be the reference against which each estimating equation will be compared. The estimating equations will be based upon serum creatinine and/or cystatin C. The eGFR-C study has 5 components:

1) A prospective longitudinal cohort study of 1300 adults with stage 3 chronic kidney disease followed for 3 years with reference (measured) GFR and test (estimated GFR [eGFR] and urinary albumin-to-creatinine ratio) measurements at baseline and 3 years. Test measurements will also be undertaken every 6 months. The study population will include a representative sample of South-Asians and African-Caribbeans. People with diabetes and proteinuria (ACR ≥30 mg/mmol) will comprise 20-30% of the study cohort.

2) A sub-study of patterns of disease progression of 375 people (125 each of Caucasian, Asian and African-Caribbean origin; in each case containing subjects at high and low risk of renal progression). Additional reference GFR measurements will be undertaken after 1 and 2 years to enable a model of disease progression and error to be built.

3) A biological variability study to establish reference change values for reference and test measures.

4) A modelling study of the performance of monitoring strategies on detecting progression, utilising estimates of accuracy, patterns of disease progression and estimates of measurement error from studies 1), 2) and 3).

5) A comprehensive cost database for each diagnostic approach will be developed to enable cost-effectiveness modelling of the optimal strategy.

The performance of the estimating equations will be evaluated by assessing bias, precision and accuracy. Data will be modelled as a linear function of time utilising all available (maximum 7) time points compared with the difference between baseline and final reference values. The percentage of participants demonstrating large error with the respective estimating equations will be compared. Predictive value of GFR estimates and albumin-to-creatinine ratio will be compared amongst subjects that do or do not show progressive kidney function decline.

**Discussion:**

The eGFR-C study will provide evidence to inform the optimal GFR estimate to be used in clinical practice.

**Trial registration:**

ISRCTN42955626.

## Background

Chronic kidney disease (CKD) is prevalent in the general population [1-4] and is commonly identified using estimation of glomerular filtration rate (GFR) or detection of protein in urine (albuminuria/proteinuria). GFR is accepted as the best overall measure of kidney function and is central to diagnosis, staging and management of CKD. Ideally GFR would be measured using reference procedures which follow the clearance of an infused exogenous substance (e.g. inulin, ^125^I-iothalamate or iohexol [5]). However, these methods are cumbersome and impractical for general kidney disease detection and management. Estimation of GFR (estimated GFR [eGFR]) using equations based on serum creatinine with adjustments for age, gender and race are widely used as surrogate measures of GFR. In England, the National Institute for Health and Care Excellence (NICE) have recommended which people should be tested for the presence of CKD (e.g. those with diabetes or hypertension) and that GFR should be estimated 6-monthly in people with stage 3 CKD (GFR 30–59 mL/min/1.73 m^2^) [6], comprising approximately 6-7% of the overall UK population [3,7]. The aim of disease detection is to identify and manage those most likely to progress to kidney failure and/or those at high risk of morbidity and mortality. In addition to the accurate identification of CKD, the ability of tests to identify which individuals with CKD will have high risk (i.e. progressive or mortal) disease is a crucial issue. Many people with stage 3 CKD are not at increased risk of progressive disease and there are concerns that CKD detection using creatinine-based approaches may be identifying individuals who are at low risk and unlikely to benefit from active management and inappropriate surveillance [8]. Recently, newer equations utilising cystatin C instead of, or in addition to, creatinine have been proposed. Given the high costs of cystatin C testing compared with creatinine, it is critical that its diagnostic accuracy and prognostic ability are carefully validated ahead of widespread introduction into the National Health Service.

### Measuring GFR

Standard clearance of inulin, including urine collection, remains the ‘gold-standard’ method for GFR measurement but few studies use this. Most evaluations of GFR equations have used radiolabelled plasma clearance methods which are assumed to be closely related to inulin clearance. Radiolabelled iothalamate plasma clearance was the method used for developing the Modification of Diet in Renal Disease (MDRD) Study [9] and Chronic Kidney Disease Epidemiology Collaboration (CKD-EPI) [10] GFR-estimating equations (see below), whilst the CKD-EPI equation validation dataset also used a variety of other reference GFR methods including iohexol [10]. Although regarded as the reference approach to assessment of kidney function, it is increasingly appreciated that non-inulin plasma clearance methods are not all equivalent [11]. Furthermore, as with any physiological measurement, GFR has an intrinsic biological variability, the understanding of which is critical to appreciation of disease-related change. Using a variety of reference markers, values (coefficient of variation, CV%) ranging between 5.5% and 11.6% have been reported for the biological variation of GFR [12-18]. However, most of these estimates were not derived using classical biological variation studies [19].

### Estimating GFR

Use of the MDRD Study equation (MDRD) [9,20,21], which estimates GFR adjusted for body surface area (BSA), has been endorsed by national professional healthcare organisations [6,22]. However, accuracy of the equation is sub-optimal. In the CKD field, accuracy of GFR estimating equations is commonly expressed as the P_30_, the percentage of eGFR values within 30% of ‘true’ GFR. This metric captures aspects of both imprecision (measurement error) and bias (systematic over- and/or under-estimation). Reported P_30_ values for the MDRD equation typically range between 73% to 93% [23]. The MDRD equation has also been criticised on the basis that it significantly underestimates GFR (particularly in individuals with GFR greater than 60 mL/min/1.73 m^2^) and has poor precision [10].

A revised equation, the Chronic Kidney Disease Epidemiology Collaboration (CKD-EPI_creatinine_) produces less biased estimates of GFR at higher levels of kidney function [10], although reportedly less accurate estimates as GFR falls below 60 mL/min/1.73 m^2^[23]. P_30_ values for the CKD-EPI_creatinine_ equation are slightly superior to those of the MDRD equation in studies that have undertaken a head-to-head comparison [23]. Cystatin C has been proposed as an improved marker of GFR compared with creatinine [24,25]. Recently, the CKD-EPI Collaboration have published two further CKD-EPI equations; one based on cystatin C (CKD-EPI_cystatin C_) and one using both cystatin C and creatinine (CKD-EPI_cystatin and creatinine C_) [26]. In the external validation datasets of Inker et al. [26] the CKD-EPI_cystatin C_ and CKD-EPI_cystatin and creatinine C_ equations achieved P_30_ values of 86% and 92% respectively. There has been little independent validation to date of these latter equations [27,28]. Amongst older people the MDRD equation achieved a P_30_ of 81% compared with 86% for the CKD-EPI_cystatin and creatinine C_ equation [27].

### Estimating GFR in British ethnic minority populations

People from South Asian and African-Caribbean backgrounds are at 3 to 5-fold increased risk of developing established renal failure compared with Caucasians. Whilst GFR-estimating equations have been validated in African-Caribbean communities from North America [29] and endemic Asian populations [30-35] there is no independent validation in British African-Caribbean populations; and no data at all amongst people originating from India, Pakistan, Sri Lanka and Bangladesh). There is some evidence that the black ethnicity coefficients in GFR estimating equations developed for use amongst African-Americans may not be transferable to other populations of African ancestry [36,37].

### Progression of kidney disease

There is no consistent definition of what constitutes renal progression in the literature. Many studies have used a doubling of serum creatinine, corresponding to an approximate halving of GFR, as an end-point defining progression, but this is insufficiently sensitive to be useful in clinical practice. Kidney Disease Improving Global Outcomes (KDIGO) have defined progression as a move to a higher disease category (e.g. stage 3A [GFR 45–59 mL/min/1.73 m^2^] to stage 3B [GFR30-44 mL/min/1.73 m^2^]) confirmed by a fall in GFR of greater than or equal to 25%, an increase in albuminuria, a greater than 25% decline in GFR (e.g. a decline from 60 mL/min/1.73 m^2^ to less than 45 mL/min/1.73 m^2^) or a greater than 10%/year decline in GFR (e.g. a decline from 60 mL/min/1.73 m^2^ to less than 54 mL/min/1.73 m^2^ in one year) [38]. NICE defined progression as a decline in GFR of more than 5 mL/min/1.73 m^2^/year, or more than 10 mL/min/1.73 m^2^ averaged over 5 years [6].

Progression is not necessarily common even amongst people with known CKD e.g. amongst people with stage 3 CKD only 1.3% progressed to stage 5 CKD (established renal failure, typically requiring dialysis or transplantation) over 5 years [39]. Amongst community dwelling older (greater than 65 years) adults with stage 3 CKD mean GFR declines of 3.6 mL/min/1.73 m^2^/year and 2.8 mL/min/1.73 m^2^/year have been reported respectively in male and female subjects with diabetes and somewhat lower values amongst subjects without diabetes (1.9 mL/min/1.73 m^2^/year and 1.1 mL/min/1.73 m^2^/year amongst males and females respectively) [40]. In the REIN study proteinuric (greater than 1 g/24 h) non-diabetic subjects with GFRs in the approximate range 30–50 mL/min/1.73 m^2^ showed a decline of GFR of 7.0 mL/min/1.73 m^2^/year with slightly lower values being observed in those receiving renin-angiotensin-aldosterone system (RAAS) blockade [41].

There are some data, mainly restricted to small studies in people with diabetes, describing disease progression in terms of decline in reference GFR measurements [42,43]. Generally, disease progression in people with diabetes has been described as following a broadly linear decline, being influenced by blood pressure and albuminuria and ameliorated by antihypertensive medication/RAAS blockade [42,44,45]. A similar pattern has been observed using estimated rather than measured GFR [46].

### Identifying progressive kidney disease

A significant problem is the ability of GFR-estimating equations to identify progression of kidney disease against background change in GFR (i.e. that due to ‘normal’ ageing; commonly cited as approximately minus 1 mL/min/1.73 m^2^/year) given the biological and measurement variability of both reference and estimated GFR. The intraindividual variation (CV_I_) of the main determinant (serum creatinine) of eGFR has been reported as 4.3% [47] to which should be added intralaboratory imprecision (CV_A_) of approximately 3.0% [22]. On this basis, the critical difference or reference change value (RCV) for serum creatinine is 13% (i.e. this is the difference that can be considered ‘real’ with 95% probability). The power function in the MDRD equation (-1.154, Table 1) increases the impact of CV_I_ to an average of 5.4%. Consequently the RCV for eGFR derived using the MDRD equation becomes 14.4%. As an example of this, in an individual a GFR of 60 mL/min/1.73 m^2^ will need to fall below 51 mL/min/1.73 m^2^ before it can be confidently considered a significant decrease. Some [48,49], although not the majority [50-53] of data suggests that the biological variation of serum cystatin C is greater than that of creatinine. If this were the case then it would clearly impact on the ability of cystatin C based GFR-estimating equations to detect changes in true GFR vis-à-vis serum creatinine.

**Table 1:** Equations to be used to estimate glomerular filtration rate (GFR)

**Abbreviation**	**GFR equation expressed as a single equation**
MDRD [20]	175 × Scr ^-1.154^ × age^-0.203^ × 0.742 [if female] × 1.212 [if black]
CKD-EPI_creatinine_[10]	141 x min(Scr/κ, 1)^α^ × max(Scr/κ, 1)^-1.209^ × 0.993^Age^ × 1.018 [if female] × 1.159 [if black], where κ is 0.7 for females and 0.9 for males, α is -0.329 for females and -0.411 for males, min indicates the minimum of Scr/κ or 1, and max indicates the maximum of Scr/κ or 1
CKD-EPI_cystatin C_[26]	133 × min(Scys/0.8, 1)^-0.499^ × max(Scys/0.8, 1)^-1.328^ × 0.996^Age^ × 0.932 [if female] where min indicates the minimum of Scr/κ or 1, and max indicates the maximum of Scr/κ or 1.
CKD-EPI_cystatin-creatinine_[26]	135 × min(Scr/κ, 1)^α^ × max(Scr/κ, 1)^-0.601^ × min(Scys/0.8, 1)^-0.375^ × max(Scys/0.8, 1)^-0.711^ × 0.995^Age^ × 0.969 [if female] × 1.08 [if black] where κ is 0.7 for females and 0.9 for males, α is -0.248 for females and -0.207 for males, min indicates the minimum of Scr/κ or 1, and max indicates the maximum of Scr/κ or 1.

*Abbreviations:* CKD-EPI Chronic Kidney Disease Epidemiology Collaboration, MDRD Modification of Diet in Renal Disease, Scr serum creatinine, Scys serum cystatin C.

GFR changes of this order exceed the limit that most nephrologists would consider a clinically insignificant change. However, there are no prospective longitudinal data assessing the relative abilities of GFR-estimating equations to detect change in underlying measured GFR. One recent study has addressed the accuracy of GFR estimating equations compared with ^125^I-iothalamate measured GFR over time in people with kidney disease [54]. The authors concluded that GFR estimating equations accurately reflected changes in measured GFR over time. The study was robust (3,532 participants with CKD followed for a mean of 2.6 years) but retrospective in nature, and did not include data derived using cystatin C [54]. Observational data suggests that for identification of progressive CKD the combination of eGFR using cystatin C and albumin-to-creatinine ratio (ACR) ranks highest, followed by eGFR using cystatin C alone, then the combination of ACR and eGFR using creatinine, and finally eGFR using creatinine alone [55].

### Objectives of the eGFR-C study

Whilst there is a significant literature describing the accuracy of creatinine-based eGFR against reference methods, there are no studies addressing the ability of GFR estimating equations, including those incorporating cystatin C, to detect change in GFR. Furthermore, there are no data addressing the accuracy of these equations in black and minority ethnic populations. The eGFR-C study will evaluate the performance of GFR-estimating equations, including novel equations incorporating cystatin C, in assessing and monitoring measured GFR in people with stage 3 CKD. The data will be analysed to assess the impact of ethnicity, proteinuria and diabetes on equation performance. eGFR-C will assess whether eGFR using either creatinine or cystatin C or a combination of both is superior at detecting changes in GFR as measured by a reference GFR method. The utility of baseline eGFR and urinary ACR to predict which people are likely to show progressive kidney disease will also be tested. A simple economic evaluation of the relative costs of the diagnostic tests will be included to enable cost-effectiveness modelling.

## Methods/design

**Figure 1:**
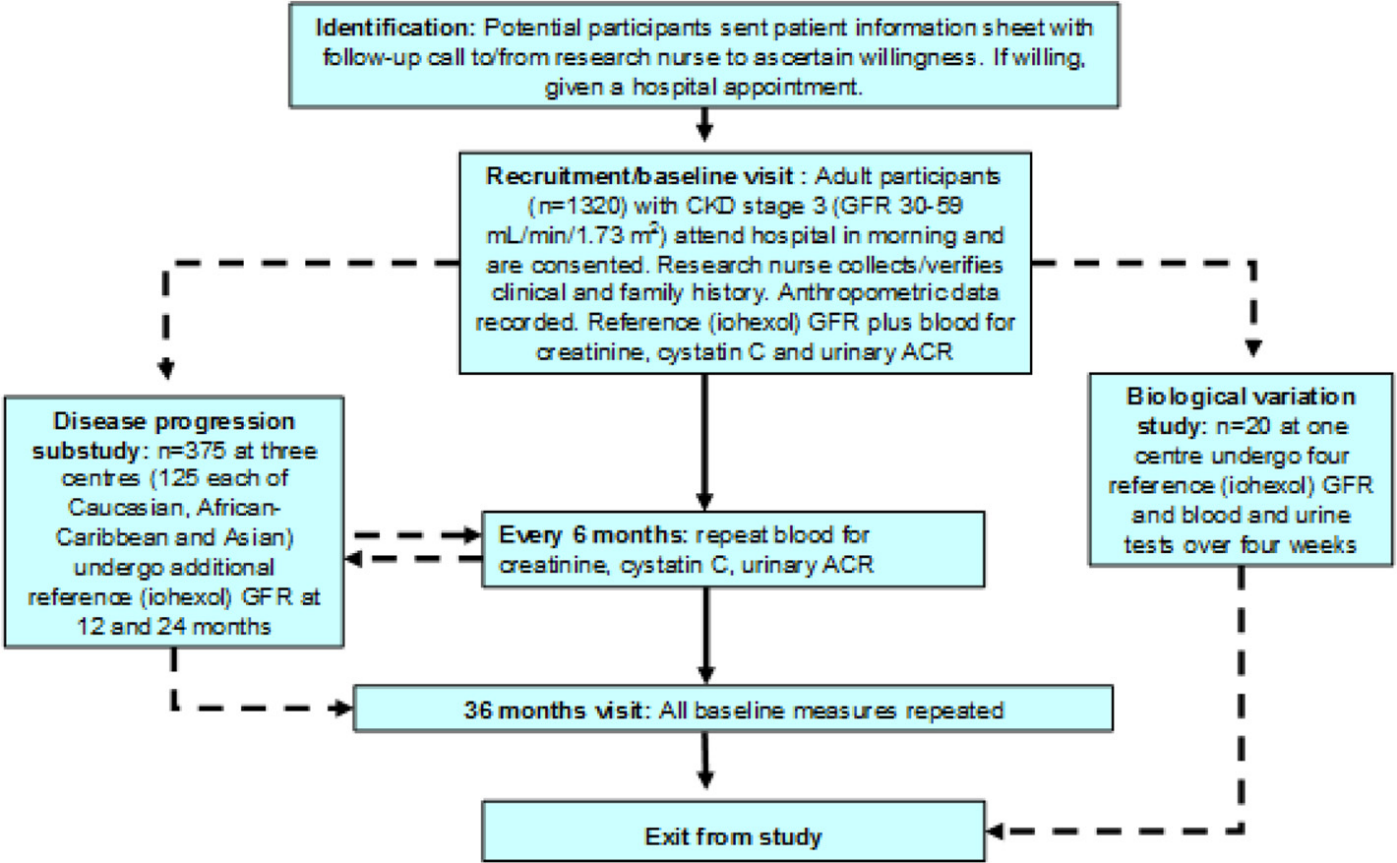
General study schema.

eGFR-C is a study which will provide the required evidence to identify the optimal estimate of GFR to use in clinical practice (Figure 1).

There are five components to the study:

1) A 3-year prospective longitudinal cohort study using 6-monthly estimates of GFR and baseline and final reference GFR values to assess and compare the accuracy and precision of each estimate of GFR and change in GFR. This will include assessments in high risk subgroups and ethnic groups.

2) Investigation and modelling of patterns of progression of GFR in a subset of the cohort, stratified by ethnicity, presence of diabetes and proteinuria, who will receive annual reference GFR, assessing risk factors and over time.

3) A sub-study investigating sources of variability to estimate the components of measurement error in each measure and estimate of GFR.

4) Using information from 1), 2) and 3) modelling of alternative monitoring strategies, using different estimates of eGFR and monitoring frequencies.

5) A model-based cost-effectiveness analysis.

### Prospective longitudinal cohort study

The primary study will comprise a prospective longitudinal cohort study in which adults (n = 1300) with stage 3 CKD, enriched to include people more likely to have progressive kidney disease (i.e. those with proteinuria and/or diabetes), will be recruited from six UK centres. Participants will be recruited from both primary (two centres) and secondary/tertiary (four centres) care. People aged 18 years and older having stage 3 CKD (eGFR measurements between 30 and 59 mL/min/1.73 m^2^ inclusive sustained over at least 3 months prior to recruitment) will be included. Recruitment will be targeted such that 20% to 30% of recruits will have proteinuria (ACR ≥30 mg/mmol) and a similar proportion will have diabetes. The prevalence of proteinuria and diabetes will be monitored during the course of the study to ensure reasonable representation.

Participants will attend hospital in the morning having been advised to consume a light breakfast (no meat or fish). A clinical (including cardiovascular) and drug history will be recorded using a standardised questionnaire. Information on ethnicity will be gathered using a modified version of the 2011 UK Census Questionnaire. Height will be measured to the nearest 0.1 cm with a rigid stadiometer. Body weight will be measured in light indoor clothing to the nearest 0.1 kg. Waist circumference will be recorded to the nearest 0.1 cm at the mid-point between the lower costal margin and the level of the anterior superior iliac crest. Hip circumference will be measured at the widest point of the hips and the maximal protrusion of the gluteal muscles. Brachial blood pressure will be measured three times in the sitting position as recommended [56]. Blood will be taken for serum creatinine, cystatin C and a urine sample will be collected for ACR. Further aliquots of serum, plasma and urine will be stored for potential analysis of future markers of GFR. An iohexol reference GFR measurement will be undertaken in addition to estimation of GFR using four GFR estimating equations (MDRD, CKD-EPI_creatinine_, CKD-EPI_cystatin_, and CKD-EPI_cystatin-creatinine_) (Table 1).

Participants will be followed for three years. All the above measurements and the clinical questionnaire will be repeated at 36 months. At each 6 month interval, all participants will have blood taken for serum creatinine, cystatin C and a urine sample will be collected for ACR. GFR will be estimated as above.

Participants will be excluded if they are pregnant, have a history of untoward reactions to iodinated contrast media, have life expectancy which makes study completion unlikely [57], are unable to consent due to cognitive impairment, are whole- or part-limb amputees, have a recent (last 6 months) episode of acute kidney injury (as defined by the Acute kidney Injury Network criteria [58]) or have sickle cell disease.

GFR will be measured using an iohexol clearance method. A 5 mL bolus of Omnipaque 240 (518 g/L iohexol corresponding to 240 g/L of iodine, GE Healthcare http://www.gelifesciences.com) followed by 10 mL of normal saline will be injected into the antecubital vein. Blood samples will be collected at 5, 120, 180 and 240 minutes after injection. Samples will be stored at -80°C prior to analysis. Iohexol will be determined using isotope dilution mass spectrometry (ID-MS) [59] and GFR calculated [60]. Serum and urinary creatinine will be measured using ID-MS and enzymatic assays respectively, both traceable to a reference methodology. Serum cystatin C will be measured using a nephelometric immunoassay with calibration traceable to the international certified reference material ERM-DA471/IFCC [61]. Urinary albumin will be measured using a nephelometric immunoassay.

Blood samples will be collected using standard venepuncture and phlebotomy procedures and samples will be transported to the local laboratory, where plasma/serum will be separated within 4 h of venepuncture. Aliquots of serum/plasma and urine will then be stored at -80°C pending transportation to the central laboratories and analysis. Clinicians and others involved in patient care will be blinded to the specific study measurements (reference [iohexol] GFR, estimated GFR and ACR) for the duration of the study.

### Sub-study of patterns of disease progression

At three centres 125 each of Caucasian, African-Caribbean and Asian subjects (i.e. n = 375 in total), stratified in each group by higher and lower risk of disease progression (approximately 60 with diabetes and/or proteinuria and 60 with neither diabetes nor proteinuria) will undergo additional sampling every year over the 3 year study period. At each point indicated subjects will undergo a reference GFR measurement in addition to cystatin C and creatinine eGFRs and ACR. The study will enable a model of disease progression to be developed based on reference GFR measurement enabling optimal monitoring frequencies in a high-risk cohort to be defined. This number of subjects should provide a range of values over the main factors considered to influence disease progression and allow assessment of covariates in the statistical model. Further assessment of covariates will be performed by combining the data from this sub-study with the main study. Inclusions/exclusions and laboratory methods for this sub-study will be as above.

### Study of intra-individual biological variability

A study will be undertaken to define the normal biological variability of a reference GFR test in addition to the eGFR tests. Twenty people with stage 3 CKD will undergo four iohexol reference measures of GFR in four successive weeks, with standardisation for time of day (morning after a light breakfast) and day of week. Inclusions/exclusions and laboratory methods for this sub-study will be as above.

### Sample size calculations

1) Prospective longitudinal cohort study

The sample size calculation for the main study focuses on the ability to detect differences in accuracy of measurement between the MDRD_creatinine_ equation and the CKD-EPI_cystatin C_ equation. We will also make secondary comparisons with the other equations**.** The measure of accuracy we will use is the P_30_. Sample size is based on a simulation study to estimate differences in P_30_ and estimates of rate of change which are not amenable to algebraic solution. Our simulation modelled the full structure of the study including random variability, and computed statistical power through noting the percentage of simulations yielding statistically significant results (P < 0.05) for analysis of each outcome. Power estimates are based on 1000 simulations.

With 1000 evaluable subjects our simulations showed the study to have 87% power at the 5% significance level to detect a difference of 5% in P_30_, between 81% and 86%, which is of a magnitude considered clinically important and likely to occur with the expected scale of differences in imprecision between the equations [27]. We thus aim to recruit 1300 people, which allowing for 15-20% drop-out will deliver over 90% power. The proposed sample size in the ethnicity subgroups will allow P_30_ estimates to be reported with 95% confidence intervals of 10 percentage points wide.

Rates of change in estimated and measured GFR will be obtained by linear regression. Padala et al. proposed two cut-points for estimating accuracy of rates of change: greater than 3 mL/min/1.73 m^2^/year error and greater than 5% error [54]. The study will have over 90% power and over 80% power at the 5% significance level to detect differences in these two outcomes respectively, for differences of a magnitude corresponding with P_30_ measures of 81% and 86%.

2) Sub-study of patterns of disease progression

A sample size of 375 was chosen based on practical considerations to allow investigation of twelve covariates of interest (gender, age, diabetes, duration of diabetes, ethnicity, albuminuria, baseline GFR, blood pressure, body mass index, waist circumference, smoking status and presence of vascular disease) in addition to variables for time, drug effects and random effects. Another consideration was to include a reasonable number of subjects in each of the ethnic groups and high and low risk subgroups to estimate disease progression.

3) Study of intra-individual biological variation

The biological variation of GFR has been estimated at approximately 10% (see above). On this assumption, the sample size (n = 20) and number of repeat samplings proposed will enable the CV_I_ to be estimated within ±2% of its true value.

### Study steering committee

The study steering committee comprises an independent chairperson (a consultant nephrologist), a second independent consultant nephrologist, a patient advocate, the study statistician, an independent statistician, the chief investigator, the study lead research nurse and one other co-investigator.

### Data analysis

#### Prospective longitudinal cohort study

Data will be analysed to address three main questions:

1. Which of the GFR-estimating equations is the most accurate assessment of reference GFR?

2. Which GFR-estimating equation most accurately reflects change in GFR?

3. Which GFR-estimating equation, together with ACR, or ACR alone, most accurately predicts those people that have progressive loss of kidney function (CKD progression)?

In each case, data will be further analysed to assess whether observed relationships amongst African-Caribbean and South-Asian subjects differ from those observed amongst Caucasians, and whether diabetes and proteinuria are predictors.

1. Which of the GFR-estimating equations is the most accurate assessment of reference GFR?

Accuracy will be assessed by establishing the proportion of GFR estimates within 30% (P_30_) of iohexol GFR, using baseline measures. P_30_ values will be compared between GFR estimating equations using McNemar’s test for paired data. Additional analysis will consider the mean and median, interquartile range and root mean square error of the distribution of differences.

2. Which GFR-estimating equation most accurately reflects change in GFR?

Rate of change in eGFR will be established by linear regression [54] utilising all available (maximum 7) eGFR time points, and will be compared with the difference between final and baseline reference GFR values. Differences in large error rates (greater than 3 mL/min/1.73 m^2^/year, or greater than 5%/year difference in slope) will be compared using McNemar’s test. Additional analyses will consider the predictive ability of the tests to detect i) a change in iohexol GFR of greater than 25%; ii) a decline in GFR of greater than or equal to 10 mL/min/1.73 m^2^ over the three years; and iii) a change that exceeds the RCV derived for the reference GFR test in the measurement variability study described below.

3. Which GFR-estimating equation, together with ACR, or ACR alone, most accurately predicts those people that have progressive loss of kidney function (CKD progression)?

Models will be constructed to predict time to progression based on baseline eGFRs and ACR. Progression will be defined in terms of decline in reference GFR (change in iohexol GFR > 10 mL/min/1.73 m^2^) or an increase in albuminuria category, as suggested by KDIGO [38]. Progression will only be detected at one of 6 time points, hence piecewise survival models will be fitted to determine whether the prognostic value of ACR and the estimated GFRs is independent of other risk factors. We will develop a prognostic model utilising age, gender, ethnicity, body mass index, waist circumference, mean arterial blood pressure, diabetes mellitus, smoking status, and presence of vascular disease in addition to baseline ACR and the various eGFRs. Both proportional and non-proportional hazards will be considered. Bootstrap validation will be used with these prediction models.

#### Sub-study of patterns of disease progression

##### **How does GFR progress over time and what are the optimal monitoring times?**

The rate of decline (mL/min/1.73 m^2^/year) in reference GFR, and the difference between reference GFR and estimated GFRs (referred to as error), measured every 12 months will be modelled over time using a longitudinal linear or nonlinear (exponential decline) random coefficients regression model to estimate average and variability in disease progression and error. Parameters of the model for each outcome will be estimated using maximum likelihood. Covariates to be explored in the model will be as described above. The effect of covariates on the population average intercept and longitudinal time effect parameters will be assessed. The method of backward elimination will be used to remove covariates which are not significant from the model. A linear relationship between disease progression and drug name or drug class will be explored. Analysis will be undertaken using NONMEM version 7.1.2, R open source software and PFIM version 3.2.2 optimal design algorithms (R open source software). The PFIM algorithms will be used to calculate the D-optimal [62] sampling times from the disease progression model based on reference GFR for individuals with diabetes and/or proteinuria, and for those individuals with neither of these conditions. Optimal monitoring strategies will be selected from a set of designs and for important subgroups of the population found to be significant in the disease progression model. The monitoring strategies identified will be used as the basis for further simulations.

#### Study of intra-individual biological variation

Pre-analytical variables will be standardised as described above. All samples for all analytes will be assayed in duplicate and the analytical variance (SD_A_^2^) will be calculated from the differences between the duplicate measurements. The total (CV_T_), analytical (CV_A_) and within-individual (CV_I_) components of variation will be calculated using nested ANOVA [19]. The critical difference (reference change value, RCV) for significant changes in serial results (P less than 0.05) and the number of specimens required to estimate the homeostatic set-point of an individual (within ±10% with a confidence of 95%) will also be estimated. The derived RCV for the reference GFR will be used to test the ability of estimated GFR equations to detect a true change in GFR (see study 1, part 2 above).

#### Modelling monitoring strategies

Whilst our longitudinal cohort will not have adequate power to detect differences in progression, our estimates of the accuracy of eGFR (study 1), patterns and determinants of progression (study 2), and intra-individual biological variation (study 3) can be combined in a model to evaluate the impact of alternative monitoring strategies on detection of progression to stage 4 CKD. True GFR values will be modelled over time for representative cohorts of people, and performance of alternative monitoring strategies in detecting progression to stage 4 CKD (varying in timing and choice of eGFR equation) will be simulated utilising estimates of measurement error and accuracy. Outcome variables which will be assessed will include false positive progression rates, and the sensitivity and delays in detecting progression.

#### Health economics study

The aim of the health economics evaluation is to determine the cost-effectiveness of implementing cystatin C-based eGFR or a combination of both cystatin C and creatinine-based eGFR in subjects that are initially stage 3 CKD compared with MDRD (creatinine-based) eGFR alone. The cost-effectiveness analysis will take the form of a cost-utility analysis in which the outcome measure will be the cost per quality adjusted life year (QALY). This will be undertaken by extending the monitoring strategy by extrapolating the rate of change in GFR beyond the end of the trial through the use of secondary data sources to link the error in estimated GFR to patient outcomes, which will include myocardial infarction, kidney transplant, and established renal failure. It is unlikely that differences in quality of life will be seen for patients receiving alternative monitoring strategies during the period of the trial, and therefore quality of life data (e.g. EQ-5D) is not being collected during this study. Instead secondary sources will be used to inform the impact of the long term outcomes of CKD on quality of life.

Cost data collection will be undertaken prospectively for all subjects in the cohort study in order to inform the cost component of the cost-effectiveness analysis. The main resource uses monitored during the trial will include the following:

1. Diagnostic testing procedures implemented.

2. Resource uses involved in the diagnostic testing procedures (e.g. urine ACR).

3. Other related procedures, including level of health care professional involvement in the procedure, equipment required, overheads, consumables etc.

The costs obtained will be dictated by the recommended investigations and interventions by GFR category in NICE Clinical Guideline 73 [6]. The difference in cost will be that between GFR category and rate of change in GFR assessed by creatinine-based estimating equations versus cystatin C-based equations.

The model-based analysis will be carried out following the conclusion of the data collection undertaken during the cohort study. A decision analytic model will be used to allow extrapolation of the cost and effectiveness parameters beyond the data observed during the trial (and to allow extrapolation to other settings). The model will consider the impact of the error in GFR measures on patient outcomes. The results of the economic analysis will be presented using cost-effectiveness acceptability curves to reflect sampling variation and uncertainties in the appropriate threshold value by which the cost-effectiveness of the different diagnostic strategies will be judged.

## Discussion

CKD is common and is usually detected using estimated GFR and/or albuminuria. Estimation of GFR on every blood creatinine request received by laboratories has been recommended by the Department of Health [63] and NICE [6]. It is estimated that more than 50 million GFR estimates are produced by UK NHS laboratories every year. Endorsement of cystatin C testing in international guidance [38] together with the increasing availability of cystatin C assays on large, automated laboratory test platforms will increase the pressure on NHS laboratories to introduce this test, which is significantly more expensive than creatinine testing. Whilst introduction of routine GFR estimations is generally deemed to have brought significant health advantages [64] there is also concern that non-diseased individuals may be identified who may undergo inappropriate investigation and surveillance [8]. Further, the ability of tests to identify which individuals with CKD will have high risk (i.e. progressive or mortal) disease is seen as a crucial issue. A significant problem has been the ability of GFR-estimating equations to identify progression of kidney disease given the biological variability of its main determinant (serum creatinine). There have been no prospective studies of the ability of GFR estimating equations to monitor progression and no studies at all of GFR estimating equations incorporating cystatin C; there have been no validations of GFR estimating equations in British ethnic minority populations. The proposed study will address these important issues.

We have chosen plasma iohexol clearance as the reference measure of GFR for our study because it is equivalent to inulin clearance, is widely used in clinical and research practice, is non-radioisotopic, can be measured accurately and precisely, and is cheap [13,65,66]. The CKD-EPI and MDRD equations will be studied because they are anchored to both creatinine and cystatin C reference methodology and therefore likely to generate data that will be valid in perpetuity. Other more recently published equations will also be evaluated (e.g. Berlin Initiative Study equations [28]). The study population will be a large cohort of people with stage 3 CKD including subjects of African-Caribbean and Asian ethnicity as well as subjects with diabetes and proteinuria. A sub-study will model disease progression in a smaller cohort. This will build on the findings of previous research, but using a prospective design with regular reference GFR measurements: the impact of medication on disease progression will be estimated and included in the model. The model will be used to define optimal sampling times for high and low risk participants, and other significant subgroups defined by the model. We will also use a classical study design to establish the biological variability of both reference and estimated GFR: this information will be used as one of the handles in defining progression and assessing the ability of GFR-estimating equations to detect it.

### Study status

The eGFR-C study commenced on 1st August 2013 and will open to recruitment in February 2014. The study co-sponsors are East Kent Hospitals University NHS Foundation Trust and University of Birmingham (Ref: RG_13-176). eGFR-C is being co-ordinated by the University of Birmingham Clinical Trials Unit. eGFR-C first received ethical approval from the South-East Coast-Surrey Research Ethics Committee of the National Research Ethics Service on 9th October 2013 (reference number 13/LO/1349). The NIHR CRN portfolio study number is 15268 and the study is registered as ISRCTN42955626. Further information may be found on the study website http://www.birmingham.ac.uk/egfr-c (accessed 18th December 2013).

## Abbreviations

ACR: Albumin to creatinine ratio; ANOVA: Analysis of variance; BSA: Body surface area; CKD: Chronic kidney disease; CKD-EPI: Chronic kidney disease-epidemiology consortium; CV: Coefficient of variation; CVA: Analytical coefficient of variation; CVI: Within-individual biological variation; CVT: Total coefficient of variation; eGFR: Estimated glomerular filtration rate; GFR: Glomerular filtration rate; ID-MS: Isotope dilution mass spectrometry; KDIGO: Kidney Disease Improving Global Outcomes; MDRD: Modification of Diet in Renal Disease; NICE: National Institute for Health and Care Excellence; RAAS: Rennin-angiotensin-aldosterone system; RCV: Reference change value.

## Competing interests

All authors declare no competing interests.

## Authors’ contributions

All authors contributed to the intellectual content and have met the following requirements: (a) significant contributions to the concept, (b) drafting or revising the article for intellectual content and (c) reading and approval of the final manuscript.

## Pre-publication history

The pre-publication history for this paper can be accessed here:

http://www.biomedcentral.com/1471-2369/15/13/prepub
